# A novel nonsense mutation in *SCAF4* associated with fliedner-zweier syndrome: a case report and review of the literature

**DOI:** 10.3389/fgene.2025.1487352

**Published:** 2025-04-11

**Authors:** Zhengfang Chen, Jing Zhao, Xiaoxuan Fan, Xiaoyan Xuan, Xiaoke Zhao

**Affiliations:** ^1^ Department of Rehabilitation, Children’s Hospital of Nanjing Medical University, Nanjing, China; ^2^ Department of Gastroenterology, Anhui Provincial Children’s Hospital, Hefei, China

**Keywords:** SCAF4 gene, fliedner-zweier syndrome, neurodevelopmental disorders, WES, RNA sequencing

## Abstract

**Introduction:**

Variants in the SR-related C-terminal domain-Associated factor 4 (SCAF4) gene are linked to Fliedner-Zweier syndrome (FZS), which presents with diverse symptoms, including mild intellectual disability, seizures, behavioral abnormalities, and various skeletal and structural anomalies. However, there is a paucity of cases describing genotypes and clinical features.

**Case presentation:**

We present the case of a 4-year and seven-month-old Chinese boy displaying intellectual impairment, language development disorder, behavioral abnormalities, and distinct facial features. Whole exome sequencing (WES) identified a heterozygous nonsense mutation, c.1693C>T (p.Arg565*), located in exon 14 of the SCAF4 gene (NM_020706). Sanger sequencing confirmed paternal inheritance of this mutation. RNA sequencing from the patient demonstrated widespread transcriptional dysregulation, reinforcing the role of SCAF4 dysfunction in impaired transcription and neurodevelopmental disorders. This mutation is novel, not previously recorded in databases such as GnomAD or dbSNP, nor reported in existing literature.

**Conclusion:**

We reviewed the clinical features of the patients reported in the literature with mutations in SCAF4 gene and described the case of a Chinese patient with this mutation. This case underscores the critical need for continued exploration of genotype-phenotype correlations, enhancing our understanding of the diverse manifestations of Fliedner-Zweier syndrome and informing future diagnostic and therapeutic strategies.

## 1 Introduction

SCAF4 is located on chromosome 21q22.11 and spans approximately 61 kilobases of genomic DNA, encoding the serine/arginine-related carboxyl-terminal domain (CTD) -associated factor 4 (Becker et al., 2008). SCAF4 is known to inhibit transcriptional reading (Rutkowski et al., 2015). Additionally, through its interaction with SCAF8, it suppresses the recognition of premature alternative polyadenylation sites, thereby preventing the excessive production of nonfunctional truncated proteins (Gregersen et al., 2019). In summary, SCAF4 plays a crucial role in transcription as an anti-termination protein, ensuring proper mRNA maturation. The initial study describing heterozygous loss-of-function (LoF) variants in the SCAF4 gene, linked to a novel autosomal dominant neurodevelopmental disorder, was first published by Fliedner et al. (Fliedner et al., 2020). This disorder is characterized by intellectual disability, seizures, behavioral abnormalities, facial dysmorphisms, and skeletal anomalies. The authors proposed that truncating or gene-disrupting variants in SCAF4 result in diverse neurodevelopmental disorders, with impaired SCAF4 function leading to altered mRNA processing and splicing. A total of four literature (Fliedner et al., 2020; Carvalho et al., 2023; Lin and Chen, 2024; Schmid et al., 2024)related to SCAF4 gene variation were retrieved from Pubmed database reviewed, a total of 68 cases of SCAF4 gene mutation have been reported in detail. In this study, we identified a new SCAF4 gene mutation through whole-exome sequencing. We reviewed the clinical phenotype of the affected patient and investigated gene expression differences via RNA sequencing to elucidate the genetic etiology.

## 2 Case description

**FIGURE 1:**
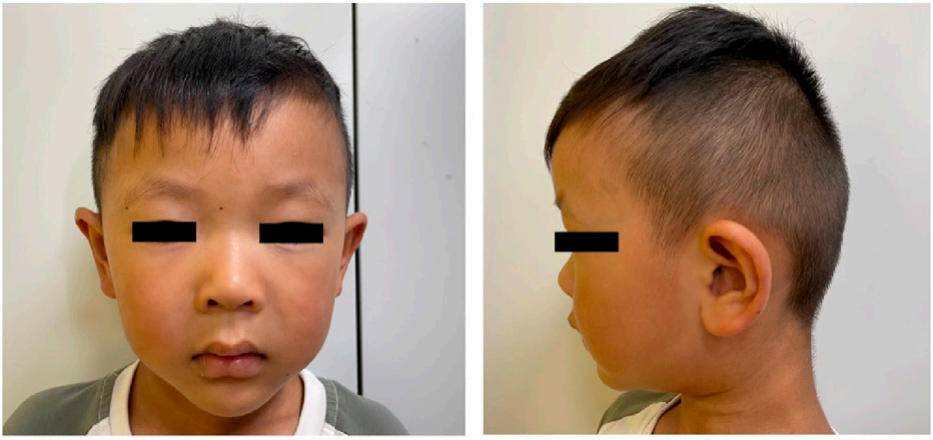
Frontal and lateral facial images of patients in this study.

The proband, a 4-year and 7-month-old boy, was admitted to the Children’s Hospital of Nanjing Medical University mainly due to language development disorder. Born full-term to non-consanguineous parents, he had no history of birth asphyxia and experienced no feeding difficulties postnatally. Apart from a paternal history of delayed language development during childhood and mild intellectual impairment, there was no relevant family history. The boy achieved independent walking at 14 months, with no significant delays in motor milestone development. A physical examination highlighted distinctive facial features, including a bulbous nose tip and elongated, slightly protruding ears (Figure 1). There was no evidence of polydactyly or syndactyly, and neurological examination revealed no positive findings. His physical development was within the normal range, with a height of 110.4 cm (75%–85%), weight of 18.3 kg (50%–75%), and a BMI of 15.1 (25%–50%). A head CT scan revealed fullness of the right lateral ventricle. Language development assessment indicated a total language ability comparable to that of a 17 to 18-month-old child, the score on the Preschool Language Scale (PLS-5)would likely be well below 70, indicating a severe language delay. Behavioral abnormalities were also observed, with parents reporting episodes of self-aggression during periods of agitation. Until his last visit, the patient had experienced no seizures and showed no skeletal or urinary abnormalities.

### 2.1 Whole exon sequencing and sanger sequencing

**FIGURE 2:**
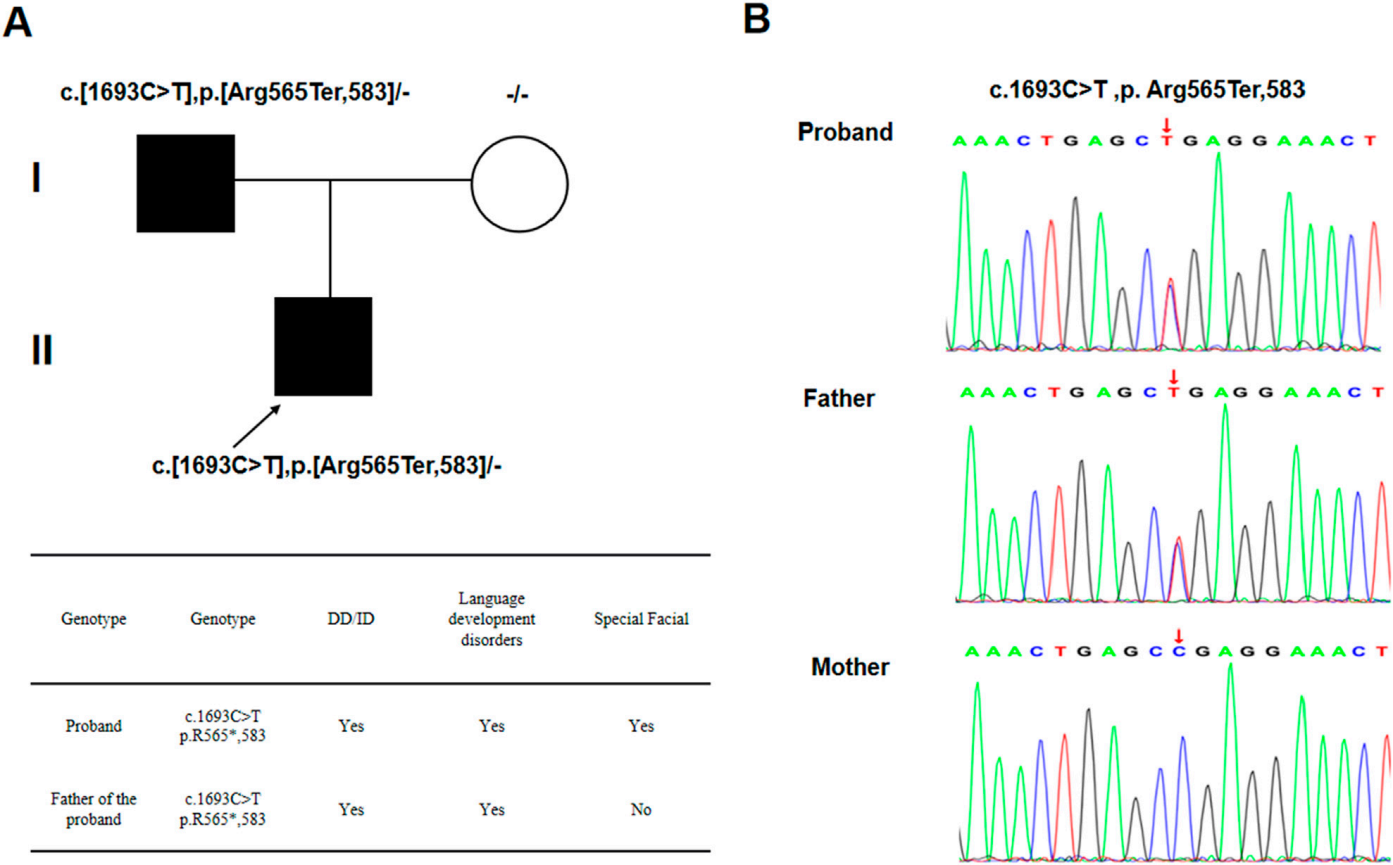
**(A)** A two-generation family map and the phenotype of the patient and his father; **(B)** Sanger sequencing results.

Whole-exome sequencing (WES) was conducted on the proband and his parents to identify the genetic cause of his condition. WES revealed a heterozygous nonsense mutation in the SCAF4 gene, c.1693C>T (p.Arg565*), located in exon 14 (NM_020706). This mutation introduces a premature stop codon, resulting in a truncated protein. Sanger sequencing confirmed that this mutation was inherited from his father, who exhibits mild symptoms (Figure 2), aligning with the mechanism of autosomal dominant inheritance (AD) disorders.

### 2.2 Bioinformatics analysis

**FIGURE 3:**
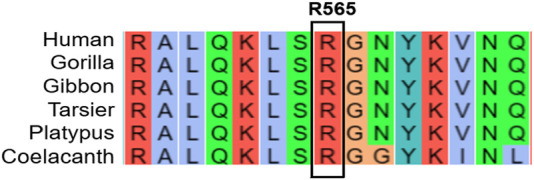
Conservative analysis of the mutated amino acid in multiple species.

According to the guidelines set by the American College of Medical Genetics and Genomics (ACMG) (Richards et al., 2015), this variant is classified as pathogenic (criteria PVS1 + PM2_Supporting + PP3). Searches in databases such as gnomAD and dbSNP revealed that this mutation has not been previously described in detail. A multi-species conservation analysis of the amino acid affected by this mutation (Figure 3) indicates that it is highly conserved across a range of species, from humans to coelacanths, highlighting its potential significance.

**FIGURE 4:**
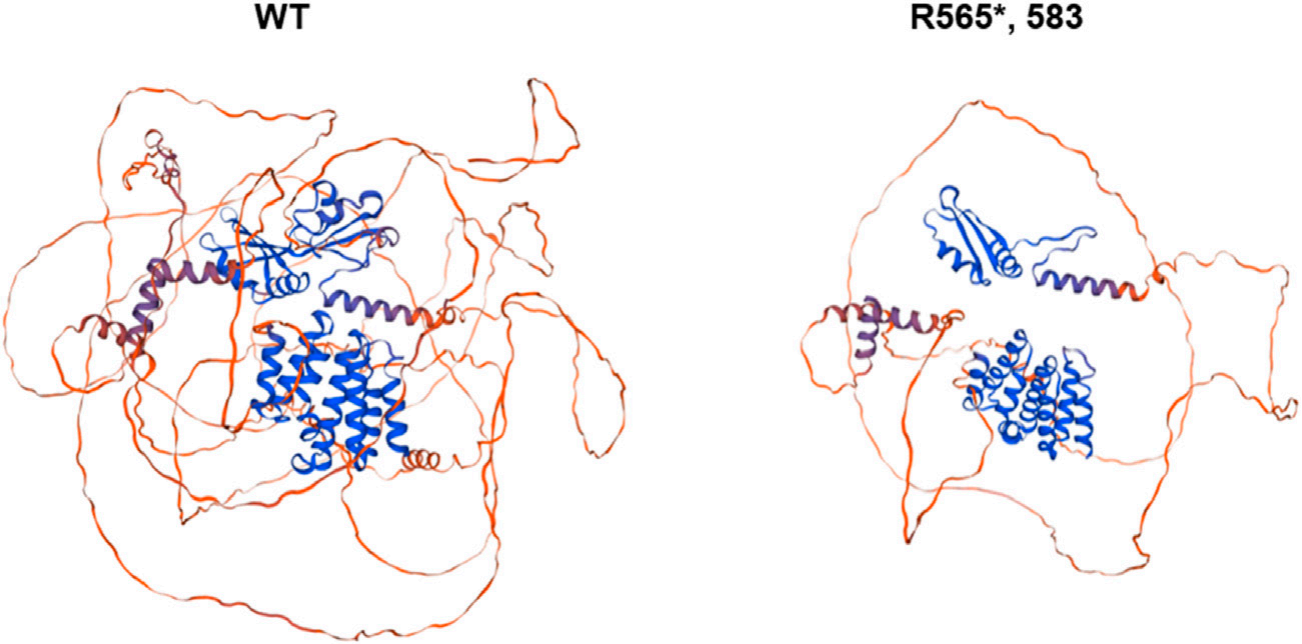
Protein modeling prediction of SCAF4 wild type (WT) and the mutant.

The SWISS-MODEL protein modeling system was used to visually predict the three-dimensional structure of wild-type mutant proteins (Figure 4). The results showed that the mutant amino acid structure was missing, which may lead to impaired protein function.

**FIGURE 5:**
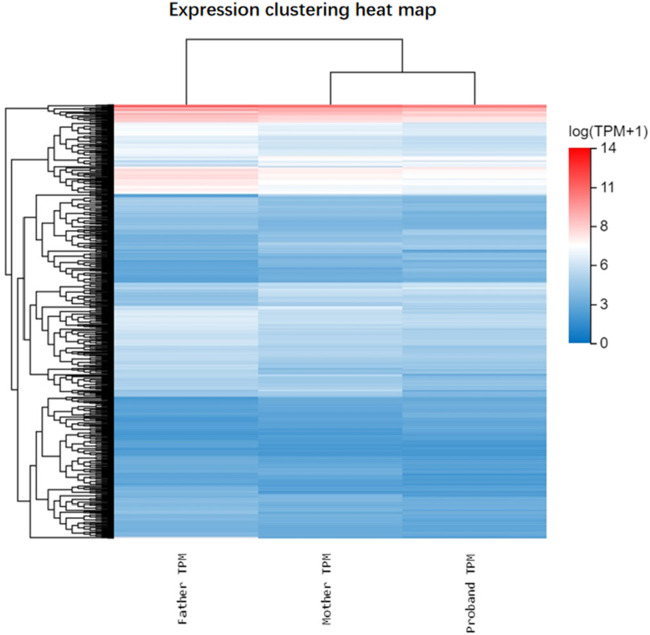
Heat maps of gene differential expression of the proband and its parents. Note: Blue, downregulated expression; Red, upregulated expression.

**FIGURE 6:**
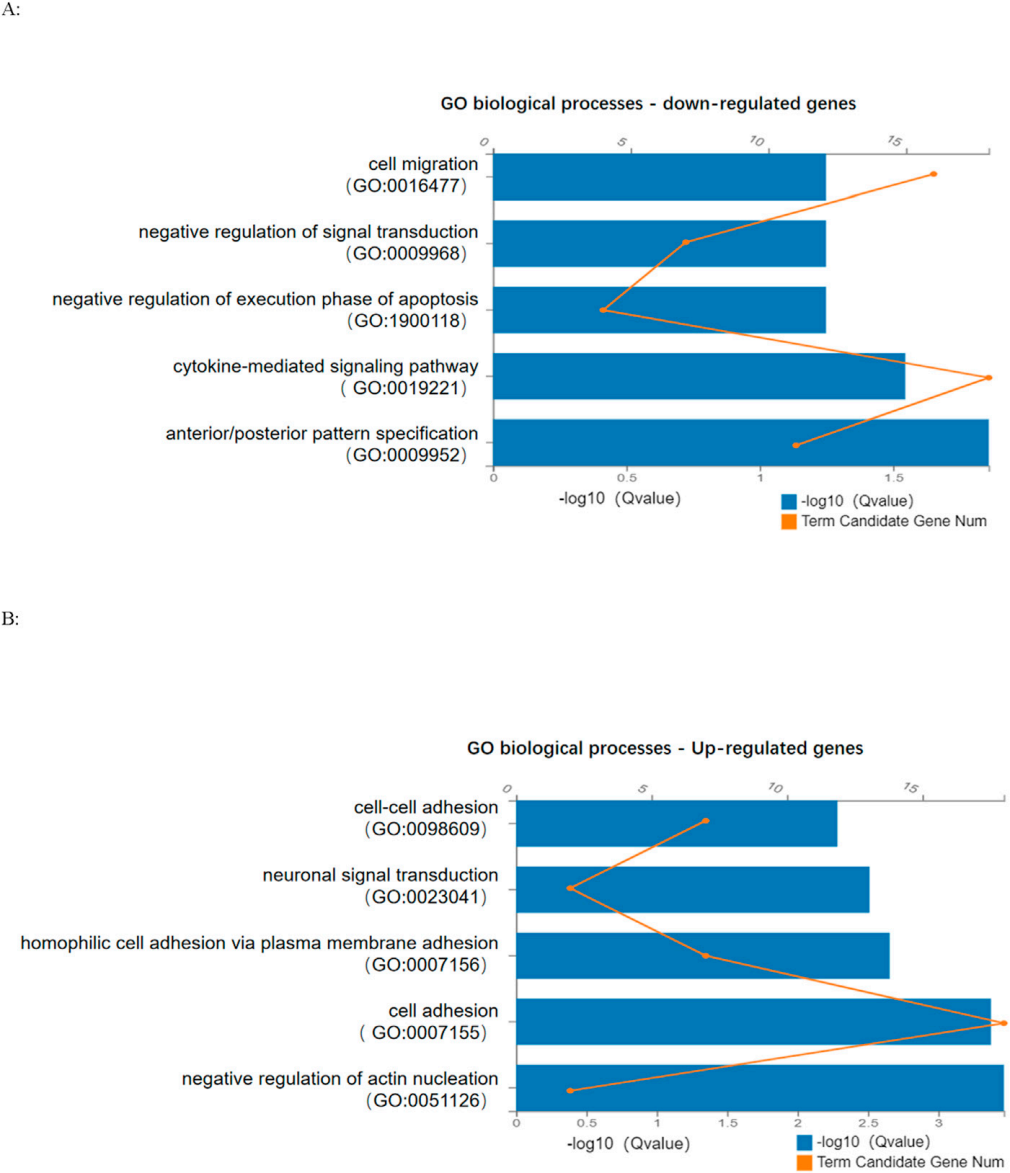
**(A)**: Enrichment term analysis of GO biological processes for downregulated genes; **(B)**: Enrichment term analysis of GO biological processes of upregulated genes.

To explore the impact of SCAF4 variants on mRNA transcription processing and termination in individual cells, we conducted paired-end RNA sequencing on peripheral blood samples from both progenitors and their parents. Our analysis identified a substantial number of differentially expressed genes between progenitors and their parents (Figure 5), reinforcing the mechanism of loss of function associated with nonsense mutations. Subsequent Gene Ontology (GO) enrichment analysis of 429 downregulated and 204 upregulated genes revealed distinct biological processes affected in the progenitor compared to the unaffected mother. The downregulated genes were significantly enriched in GO terms related to the negative regulation of cell migration and signal transduction (Figure 6A). Conversely, upregulated genes were predominantly associated with biological processes such as neuronal signaling and intercellular adhesion (Figure 6B).

### 2.3 Quantitative real-time PCR

**FIGURE 7:**
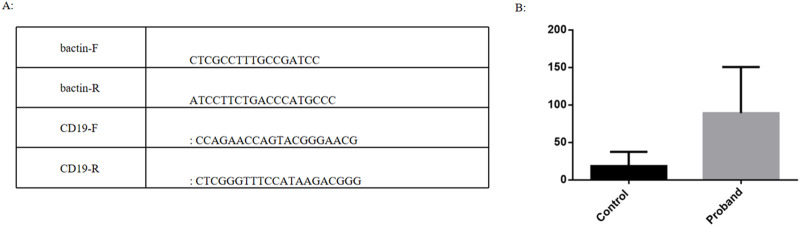
**(A)**: Primer design; **(B)**: Data analysis.

Quantitative Polymerase Chain Reaction (QPCR) is one of the commonly used techniques for quantifying nucleic acid molecules in biological and environmental samples. Changes in fluorescence signals are used to monitor the changes in product quantity after each cycle of PCR amplification in real time. The initial template was quantitatively analyzed by the relationship between Ct value and standard curve. The main procedures include RNA extraction, reverse transcription, primers design, Q-PCR and data analysis. We extracted the blood of patient and three children of the same age and sex for RNA extraction and reverse transcription. Some genes with significantly changed expression levels were selected from the results of RNA sequencing and verified by QPCR technology. It was found that the expression of CD19 in patients was significantly upregulated compared with children of the same age and sex, which was consistent with sequencing (Figure 7).

## 3 Literature review

**FIGURE 8:**
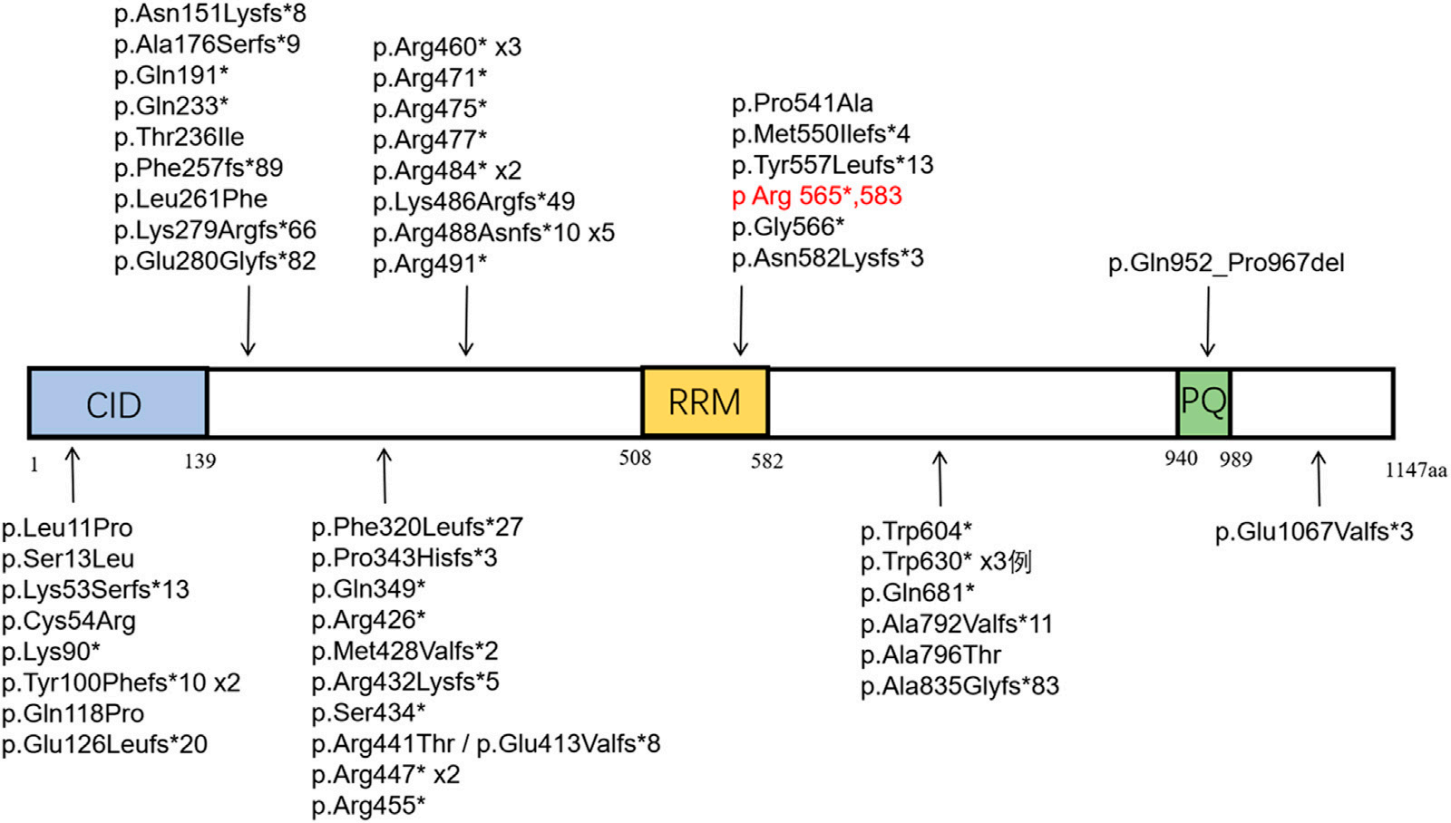
Distribution of reported SCAF4 variants on SCAF4 protein. (Note: Black represents the variation reported in the literature, red represents the variation in this study.

A total of five literature related to SCAF4 gene variation were retrieved from Pubmed database. We conducted a systematic review of the reported SCAF4 gene variants and associated phenotypes. To date, 68 cases of SCAF4 gene mutations have been documented, including two case with a complex heterozygous mutation, comprising 70 mutation sites (Supplementary Table S1). The main types of mutations include frameshift mutations (26/70, 37%) and nonsense mutations (24/70, 34%). Additionally, there are splice-site mutations (8/70, 11%), missense mutations (9/70, 13%), as well as small deletions and duplications (3/70, 4%). Most mutations in the 68 patients are *de novo* (38/68, 56%), with a predominance of male patients (45/68, 66%). The age of onset ranges from 20 months to 47 years, with the vast majority of patients experiencing onset before the age of 18 (59/68, 87%). The mutation sites reported in the literature and identified in our study were mapped onto the SCAF4 gene and protein structure (Figure 8).

The phenotypes of reported cases were also summarized (Table 1). Based on our literature review, onset ages ranged from 20 months to 47 years, with a notable male predominance (45 males to 23 females). More than 85% patients with SCAF4 gene variants exhibited developmental delays or intellectual impairments, with language development disorders being particularly pronounced. Language development disorders were observed in 46 patients, behavioral abnormalities such as hyperactivity, autism spectrum disorder, and aggressive behavior were common comorbidities. Distinct facial features such as epicanthus, a deep nasal bridge, a bulbous nose tip, and a long midface were identified in 38 patient. 30 patients experienced seizures, primarily focal seizures, which responded well to antiepileptic medications. In these 30 patients, including 11 nonsense mutations, 10 frameshift mutations, five missense mutations, and four splicing mutation. Notably, distributions can be seen in all domains, but mainly in the disordered region and CID region. Additionally, 31 patients exhibited skeletal system abnormalities, eight had cardiovascular system abnormalities, and eight had renal system abnormalities. Consistent with previous findings, the patient in this study showed language development disorders, mild intellectual impairment, behavioral abnormalities and facial anomalies. However, unlike other cases, this patient had not experienced seizures up to the last follow-up.

**TABLE 1:** The main clinical phenotype statistics of 68 patients with SCAF4 gene mutations.

Genotype	Yes	No	NA	Our patient
DD/ID	59	7	2	Yes
Language development disorders	46	10	12	Yes
Special Facial	38	19	11	Yes
Epilepsy	35	30	3	No
Skeletal abnormality	31	27	10	No
Cardiovascular abnormality	8	37	23	No
Renal abnormality	8	38	22	No

Note: The main clinical phenotype statistics of 68 patients with SCAF4 gene mutations, NA: not applicable, DD/ID: Developmental delay and/or intellectual disability.

## 4 Discussion

SCAF4 is composed of 20 exons encoding SR (serine and arginine) related CTD (C-terminal domain) related factor 4, which is composed of 1,147 amino acids and contains a CTD interaction domain (CID) and RNA recognition motif (RRM) domain. It is an SR-like CTD related factor rich in arginine/serine, as well as an important paralogue SCAF8 (Becker et al., 2008). In eukaryotes, RNA polymerase II(RNAP II) is involved in the transcription of protein-coding genes, its regulation is largely dependent on the C-terminal domain (CTD) on RNAP II’s largest subunit, RPB1(Yahia et al., 2023). CTD consists of heptaeptide repeats containing the shared sequence Tyr1-SER2-Pro3-THR4-SER5-PRO6-ser7, which is highly conserved in evolution and is critical for mRNA biogenesis and correct transcriptional termination (Eick and Geyer, 2013; Lenasi and Barboric, 2013). During transcription, SCAF4 and SCAF8 function as mRNA anti-terminators, preventing the production of non-functional truncated proteins by inhibiting the premature use of proximal and alternative polyadenylation (polyA) sites and the early processing of the mRNA 3′ end. However, their roles diverge in transcriptional regulation: SCAF8 acts as a positive elongation factor, promoting the efficient elongation of RNAP II transcription, while SCAF4 serves to limit transcriptional read-through across numerous genes (Gregersen et al., 2019).

Fliedner et al. (2020) were the first to report Fliedner-Zweier syndrome (FZS), a neurodevelopmental disorder caused by heterozygous mutations in the SCAF4 gene located on chromosome 21q22 in 2020. FZS is characterized by a spectrum of clinical features, including mild intellectual disability, seizures, behavioral abnormalities, and various skeletal and structural anomalies. FZS is an exceptionally rare condition, as of the submission date of this paper, five publications discussing SCAF4 gene variations are available. These reports document a total of 68 cases of SCAF4 gene mutations, including 2 case with a complex heterozygous mutation, encompassing 70 mutation sites in total. The patient reported in this study represents the 69th case of SCAF4 gene mutation worldwide and the seventh case identified in the Chinese population.

Almost all patients with SCAF4 gene variants exhibit developmental delay/intellectual impairment, mainly Language development disorders, behavioral abnormalities such as autism spectrum disorders, hyperactivity, and aggressive behavior. The age of onset varies and most cases are reported in males. Additionally, multiple systems, including the skeletal, cardiovascular, and renal systems, may be affected. 85% of pathogenic/potentially pathogenic variation in SCAF4 gene reported in the literature lead to early termination of protein translation. Fliedner et al. (2020) demonstrated that knocking out the SCAF4 homolog CG4266 in a *Drosophila* model results in impaired motor function, learning deficits, and short-term memory loss. Furthermore, RNA sequencing of peripheral blood from individuals with SCAF4 variants revealed impaired mRNA processing and widespread transcriptional dysregulation. The nonsense-mediated mRNA decay (NMD) (Patro et al., 2023) mechanism and the loss of function due to an insufficient haploid dose of truncated variants are proposed as the genetic causes of developmental delay and intellectual impairment in neurodevelopmental disorders. In our study, we performed paired-end RNA sequencing on peripheral blood from affected individuals and their parents. A large number of genes were differentially expressed between the progenitors and his parents, supporting the hypothesis that nonsense mutations in SCAF4 lead to loss of function and are associated with transcriptional impairment and neurodevelopmental disorders. Q-PCR detection showed that the expression of CD19 in patients was significantly upregulated compared with children of the same age and gender, which was consistent with the results of RNA sequencing. While the limited sample size introduces some variability to the results, this study contributes to the growing evidence linking SCAF4 gene dysfunction to neurodevelopmental impairment.

Previous studies have suggested a potential link between transcription-related gene variants and epilepsy (Evans et al., 2022). SCAF4 has been shown to function as an anti-terminator in the transcription process (Rutkowski et al., 2015; Gregersen et al., 2019). Hu et al. (2024) discovered that most truncated variants were associated with epilepsy. In SCAF4 knockout zebrafish models, abnormal epileptic activity and bone development issues were observed. In the 30 cases of epilepsy, the predominant types of mutations involved are nonsense mutations, frameshift mutations and splice-site mutations. Among these, more than 83% have mutations leading to premature stop codons and truncated proteins. These mutations are distributed across various domains but are mainly located in the disordered region (17/30) and the CID region (7/30). 78% mutations in the CID region are associated with epilepsy, suggesting a potential link between mutations in the CID region and epilepsy. The patient carries a variant in the RRM region of the SCAF4 subunit (p.Arg565*583). Although no seizures have been observed so far, it is important to monitor for the potential development of epilepsy in the future.

In summary, we identified a proband carrying a SCAF4 nonsense mutation, presenting with a neurodevelopmental disorder primarily characterized by language development delay. Sanger sequencing results support an autosomal dominant inheritance mechanism for the pathogenic SCAF4 variant. Current evidence suggests that heterozygous loss-of-function variants in SCAF4 can impair mRNA processing, leading to neurodevelopmental disorders. However, further studies with larger sample sizes are needed to elucidate the underlying pathogenesis and the relationship between clinical phenotypes and SCAF4 mutations. Clinically, managing patients with SCAF4 gene variants should include careful monitoring for multisystem involvement, with particular attention to the potential development of epilepsy.

## Data Availability

Publicly available datasets were analyzed in this study. This data can be found here: no.

## References

[B1] Becker R. Loll B. Meinhart A. (2008). Snapshots of the RNA processing factor SCAF8 bound to different phosphorylated forms of the carboxyl-terminal domain of RNA polymerase II. J. Biol. Chem. 283 (33), 22659–22669. 10.1074/jbc.M803540200 18550522

[B2] Carvalho L. Pinto C. F. de Oliveira S. M. Otto P. A. Krepischi A. Rosenberg C. (2023). SCAF4-related syndromic intellectual disability. Am. J. Med. Genet. A 191 (2), 570–574. 10.1002/ajmg.a.63032 36333968

[B3] Eick D. Geyer M. (2013). The RNA polymerase II carboxy-terminal domain (CTD) code. Chem. Rev. 113 (11), 8456–8490. 10.1021/cr400071f 23952966

[B4] Evans D. R. Qiao Y. Trost B. Calli K. Martell S. Jones S. (2022). Complex autism spectrum disorder with epilepsy, strabismus and self-injurious behaviors in a patient with a *de novo* heterozygous POLR2A variant. Genes (Basel) 13 (3), 470. 10.3390/genes13030470 35328024 PMC8955435

[B5] Fliedner A. Kirchner P. Wiesener A. van de Beek I. Waisfisz Q. van Haelst M. (2020). Variants in SCAF4 cause a neurodevelopmental disorder and are associated with impaired mRNA processing. Am. J. Hum. Genet. 107 (3), 544–554. 10.1016/j.ajhg.2020.06.019 32730804 PMC7477272

[B6] Gregersen L. H. Mitter R. Ugalde A. P. Nojima T. Proudfoot N. J. Agami R. (2019). SCAF4 and SCAF8, mRNA anti-terminator proteins. Cell 177 (7), 1797–1813.e18. 10.1016/j.cell.2019.04.038 31104839 PMC6579486

[B7] Hu Y. Zhang B. Chen L. He J. Yang L. Chen X. (2024). SCAF4 variants are associated with epilepsy with neurodevelopmental disorders. Seizure 116, 116113–116118. 10.1016/j.seizure.2023.10.008 37891035

[B8] Lenasi T. Barboric M. (2013). Mutual relationships between transcription and pre-mRNA processing in the synthesis of mRNA. Wiley Interdiscip. Rev. RNA 4 (2), 139–154. 10.1002/wrna.1148 23184646

[B9] Lin H. Chen Y. H. (2024). SCAF4 variants associated with focal epilepsy accompanied by multisystem disorders. Seizure 116, 11665–11673. 10.1016/j.seizure.2023.06.018 37394306

[B10] Patro I. Sahoo A. Nayak B. R. Das R. Majumder S. Panigrahi G. K. (2023). Nonsense-mediated mRNA decay: mechanistic insights and physiological significance. Mol. Biotechnol. 66, 3077–3091. 10.1007/s12033-023-00927-4 37930508

[B11] Richards S. Aziz N. Bale S. Bick D. Das S. Gastier-Foster J. (2015). Standards and guidelines for the interpretation of sequence variants: a joint consensus recommendation of the American College of medical genetics and genomics and the association for molecular pathology. Genet. Med. 17 (5), 405–424. 10.1038/gim.2015.30 25741868 PMC4544753

[B12] Rutkowski A. J. Erhard F. L'Hernault A. Bonfert T. Schilhabel M. Crump C. (2015). Widespread disruption of host transcription termination in HSV-1 infection. Nat. Commun. 67126. 10.1038/ncomms8126 PMC4441252 25989971

[B13] Schmid C. M. Gregor A. Ruiz A. Manso B. C. Herman I. Ammouri F. (2024). Further delineation of the SCAF4-associated neurodevelopmental disorder. Eur. J. Hum. Genet. 10.1038/s41431-024-01760-2 PMC12048650 39668183

[B14] Yahia Y. Pigeot A. EL A. A. Shah N. Karasu N. Forne I. (2023). RNA polymerase II CTD is dispensable for transcription and required for termination in human cells. EMBO Rep. 24 (9), e56150. 10.15252/embr.202256150 37424514 PMC10481650

